# The association between the neutrophil-to-lymphocyte ratio, platelet-to-lymphocyte ratio, and lymphocyte-to-monocyte ratio and delirium in ischemic stroke patients

**DOI:** 10.3389/fmed.2024.1456742

**Published:** 2025-01-06

**Authors:** Pangbo Wang, Jing Huang, Liwei Xu, Rong Hu

**Affiliations:** ^1^State Key Laboratory of Trauma, Burn, and Combined Injury, Chongqing Key Laboratory of Precision Neuromedicine and Neuroregenaration, Department of Neurosurgery, Southwest Hospital, Third Military Medical University (Army Medical University), Chongqing, China; ^2^Department of Trauma Neurosurgery, NO. 946 Hospital of PLA Land Force, Yining, China; ^3^School of Nursing, Peking University, Beijing, China; ^4^Department of Burn Plastic Surgery, NO. 946 Hospital of PLA Land Force, Yining, China

**Keywords:** inflammation, ischemic stroke, Mendelian randomization, risk factor, delirium

## Abstract

**Background:**

Delirium is a severe neuropsychiatric symptom following acute ischemic stroke (IS) and is associated with poor outcomes. Systemic inflammation and immune dysregulation are believed to contribute to the pathophysiology of delirium. The neutrophil-to-lymphocyte ratio (NLR), platelet-to-lymphocyte ratio (PLR), and lymphocyte-to-monocyte ratio (LMR) are widely recognized as convenient and reliable biomarkers of systemic inflammation. However, their association with delirium after IS remains unclear.

**Methods:**

In this study, we identified IS patients requiring ICU admission from the Medical Information Mart for Intensive Care (MIMIC)-IV database. We employed multivariable logistic regression and restricted cubic splines (RCS) to assess the association between the NLR, PLR, and LMR and delirium. Two-sample Mendelian randomization (MR) analysis was performed to further explore their causal relationship at the genetic level.

**Results:**

A total of 1,436 patients with IS were included in this study, of whom 214 (14.9%) had delirium. In the multivariate logistic regression analysis, after adjustment for confounders, the patients in the highest quartile of the NLR (odds ratio [OR] 2.080, 95% confidence interval [CI], 1.282–3.375) and LMR (OR 0.503, 95% CI 0.317–0.798) and the patients in the second quartile of the PLR (OR 1.574, 95% CI 1.019–2.431) were significantly associated with delirium. The RCS function showed a progressive increase in the risk of delirium with higher NLR and PLR and lower LMR. In the MR analysis, only the PLR was negatively associated with the risk of delirium.

**Conclusion:**

The observational studies found significant associations between the NLR, PLR, and LMR and delirium. However, the MR analysis only demonstrated a potential protective causal relationship between the PLR and delirium. Further prospective studies are needed to validate their association and to elucidate the underlying mechanisms.

## 1 Introduction

According to the World Health Organization, stroke is the second leading cause of death and the third leading cause of disability worldwide (1). As one of the most devastating neurological disorders, stroke imposes a significant economic and healthcare burden on society (2). Stroke can be divided into two major categories based on neuropathology: ischemic stroke (IS) and hemorrhagic stroke (3). Among them, IS is characterized by the interruption of cerebral blood supply due to various causes, resulting in corresponding neurological impairment, and it accounts for approximately 70–80% of all strokes (4). Intracranial atherosclerotic plaques and large artery stenosis are identified in 45–62% of patients with IS (5), while microvascular thrombosis and small artery occlusion are responsible for 25% of cases (5) and cardiogenic embolism is attributed to IS in 22% of cases (6, 7). While the neurological function of the majority of IS patients improves after intravenous thrombolysis or mechanical thrombectomy, some still suffer from varying degrees of complications, such as delirium and speech disorders (8).

Delirium is a severe neuropsychiatric symptom commonly observed in elderly patients following acute illness or surgery, characterized by a rapid decline in cognitive function, impaired attention, and disturbance of consciousness (9, 10). It is a common complication in hospitalized patients, with an incidence of approximately 15 to 27% in patients with stroke (11–14). Delirium has been associated with prolonged hospital stays (15), increased in-hospital mortality (16), permanent cognitive impairment (17, 18), and dementia (19) in patients with IS.

However, the pathophysiological mechanisms underlying delirium remain unclear, and there are currently no effective intervention measures available in clinical practice. Therefore, it is particularly important to identify modifiable risk factors and establish intervention strategies. Previous observational studies have suggested that inflammatory factors may be one of the precipitating factors for delirium (20, 21). Among the various inflammatory markers, the neutrophil-to-lymphocyte ratio (NLR), platelet-to-lymphocyte ratio (PLR), and lymphocyte-to-monocyte ratio (LMR) have emerged as convenient and reliable indicators of systemic inflammation. The NLR reflects the balance between the innate immune response (mediated by neutrophils) and adaptive immunity (mediated by lymphocytes), serving as a proxy for immune system dysregulation. Similarly, the PLR captures the interplay between inflammation and thrombophilia, while the LMR provides insight into the severity of systemic inflammation through its negative correlation with inflammatory activity. These readily available biomarkers have shown promise in predicting outcomes in various inflammatory and cardiovascular conditions (22), yet their roles in delirium following IS remain underexplored.

In this study, we specifically focused on ischemic stroke (IS) patients requiring ICU admission. We aimed to investigate the relationship between the NLR, PLR, and LMR and post-IS delirium, with the goal of identifying potential biomarkers for the early prediction of delirium. To achieve this, we combined observational data from the Medical Information Mart for Intensive Care (MIMIC)-IV database with Mendelian randomization (MR) methods. MR is an emerging epidemiological approach that uses genetic variables as proxies to assess the effects of exposures (e.g., NLR, PLR, and LMR) on specific outcomes (e.g., delirium) (23). Compared to observational studies, this approach can avoid the effects of confounding and reverse causality and can better simulate the results of randomized controlled trials. In this study, we combined observational data from the MIMIC-IV database with MR methods to investigate the causal effects of the NLR, PLR, and LMR on delirium after IS.

## 2 Methods

### 2.1 Overall study design

This study consisted of two phases. In the first phase, we performed multivariable logistic regression analysis using data from the MIMIC-IV v2.2 database to explore the association between the NLR, PLR, and LMR and delirium after IS. Before using the MIMIC database, we obtained institutional review board approval from Beth Israel Deaconess Medical Center and the Massachusetts Institute of Technology (record ID: 12299215).

In the second phase, we further investigated the causal relationship between the NLR, PLR, and LMR and delirium using summary statistics from genome-wide association studies (GWAS) through MR analysis. Based on the law of independent assortment, genetic variations selected as instrumental variables (IVs) for the exposure are randomly allocated to gametes during meiosis (24). Due to the random assignment of genotypes, MR analysis using single nucleotide polymorphisms (SNPs) as genetic IVs can effectively mimic the design of a randomized controlled trial (RCT), enabling the estimation of causal effects between the exposure and outcome without being influenced by traditional confounders (25), such as sex, since genetic variations do not systematically vary with demographic factors. The GWAS data for delirium were obtained from the ninth version of the FinnGen database, which includes 3,039 cases and 356,660 controls (ID: F5_DELIRIUM). According to the database, all cases of delirium were classified as conditions not caused by alcohol or other psychoactive substances. The GWAS data for the NLR, PLR, and LMR were obtained from a large-scale analysis of leukocyte-related genetic data, as published in the study by Zhou et al. (26). This study utilized summary statistics derived from the UK Biobank, and the datasets are publicly available. The specific details of the GWAS data are as follows: the GWAS summary statistics for the NLR were obtained from the study by Zhou et al. (26). The analysis included 5,973 SNPs and was conducted on a population of European ancestry, as described by the authors. The GWAS summary statistics for the PLR were sourced from the same study (26), involving 15,473 SNPs and based on a cohort of European ancestry. The data for the LMR were also obtained from the study by Zhou et al. (26), with analysis including 4,606 SNPs, conducted on a population of European ancestry, as described by the original research group. To obtain effective causal estimates, the IVs used in MR analysis must meet the following criteria: (1) Genetic variations must be strongly associated with the levels of the NLR, PLR, and LMR; (2) Genetic variations should not be influenced by confounding factors; (3) Genetic variations should affect delirium only through their influence on the NLR, PLR, and LMR. In addition, these summary statistic data come from different sample databases of European ancestry, and the probability of sample overlap is small. The original research for these GWAS studies had already received ethics committee approval (27, 28). Therefore, this study did not require additional ethical review.

### 2.2 Data sources and patient selection

**Figure 1:**
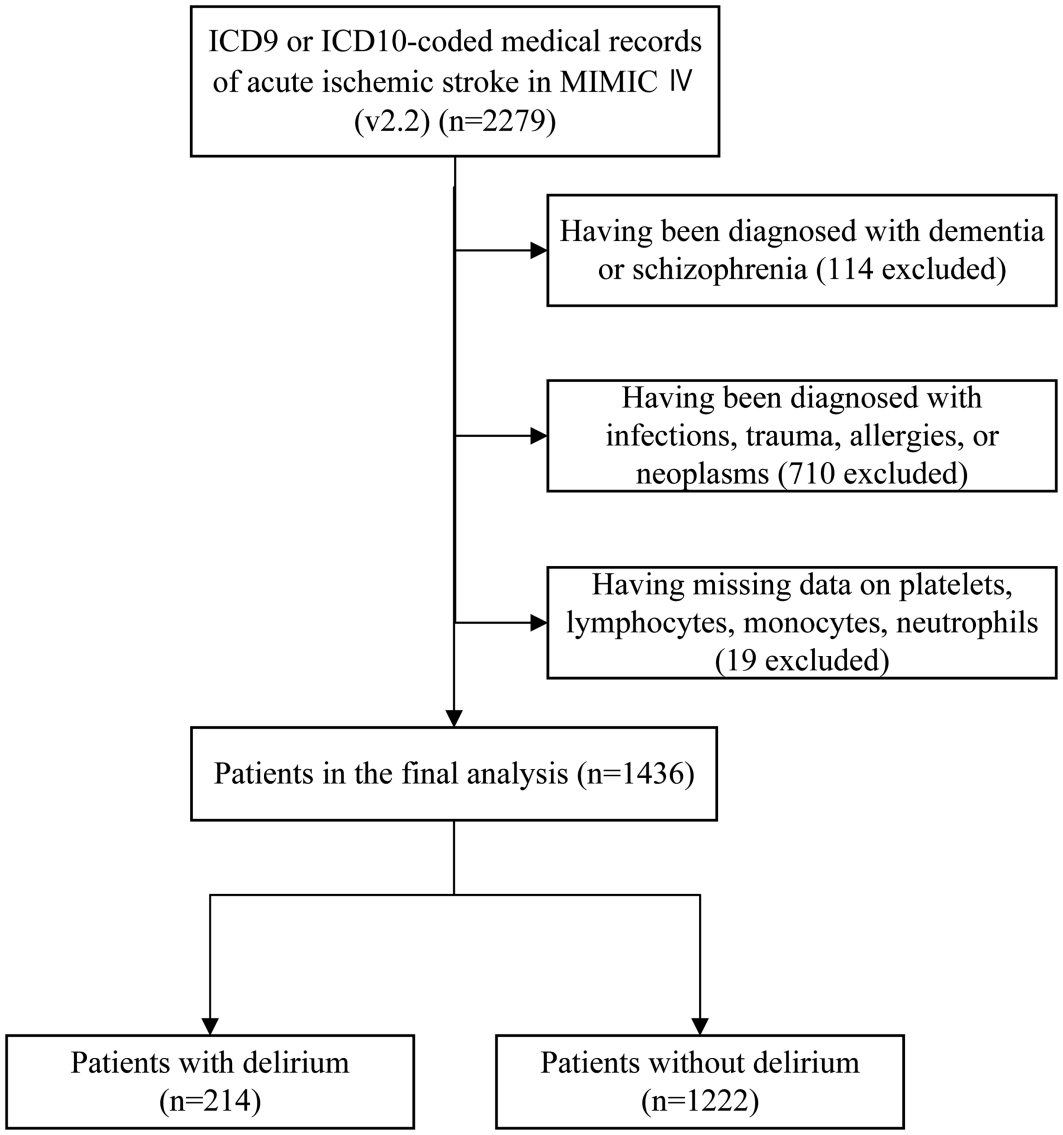
Flow diagram of patient selection.

This study used data from the MIMIC-IV (version 2.2) database, a comprehensive clinical database containing records of patients admitted to the intensive care units (ICUs) of Beth Israel Deaconess Medical Center. The MIMIC-IV database was developed and is managed by the MIT Laboratory for Computational Physiology. Patients diagnosed with IS according to the International Classification of Diseases, 9th and 10th revisions, were included in this study. The primary outcome of this study was to determine the presence of delirium during hospitalization. The assessment of delirium was primarily conducted using the widely adopted clinical tool, the Confusion Assessment Method for the ICU (CAM-ICU). The CAM-ICU consists of four features: Feature 1: acute changes or fluctuations in mental status; Feature 2: inattention; Feature 3: disorganized thinking; and Feature 4: altered level of consciousness (LOC) (29, 30). A patient was considered CAM-ICU-positive and diagnosed with delirium when Features 1 and 2 were both present, along with either Feature 3 or Feature 4 (29). The exclusion criteria were as follows: (1) patients who were admitted to the ICU multiple times for IS, with only the record of the first admission being extracted; (2) patients without sufficient data from the NLR, PLR, and LMR examinations on the first day of their stay in the ICU; (3) patients with a history of schizophrenia; and (4) patients with dementia, infections, trauma, allergies, or neoplasms. Ultimately, a total of 1,436 patients were enrolled in this study and categorized into two groups based on the presence or absence of delirium (Figure 1).

### 2.3 Data extraction

These data were extracted from the MIMIC-IV database using Structured Query Language (SQL) and PostgreSQL (version 13.7.2) software. The dataset included the following information: (1) demographic characteristics: age, sex, ethnicity, body mass index (BMI), and adverse lifestyle factors; (2) comorbidities: diabetes, hypertension, heart disease, chronic kidney disease, chronic lung disease, chronic liver disease, peripheral vascular disease, and obstructive sleep apnea; (3) laboratory parameters: hemoglobin, platelet count, serum creatinine, fasting blood glucose, serum albumin, white blood cell count, lymphocyte count, monocyte count, and neutrophil count; (4) admission severity scores: the Sequential Organ Failure Assessment (SOFA) score and Glasgow Coma Scale (GCS) score; (5) admission vital signs: heart rate, mean arterial pressure (MAP), respiratory rate, and peripheral oxygen saturation (SpO_2_); (6) use of benzodiazepines; (7) and outcomes: the primary outcome was the onset of delirium, and the secondary outcomes included length of hospital stay and in-hospital mortality. The NLR, PLR, and LMR were calculated using the following formulas: (1) NLR = neutrophil count/lymphocyte count, (2) PLR = platelet count/lymphocyte count, and (3) LMR = lymphocyte count/monocyte count. The ICD-9 and ICD-10 codes used to identify IS and delirium are shown in Supplementary Table S1.

### 2.4 Statistical analysis

For the observational studies using MIMIC-IV data, categorical variables were presented as numbers (n) and percentages (%), while continuous variables were presented as median (interquartile range, IQR) due to their non-normal distribution. The chi-squared test was used to compare the categorical variables between the groups. For the continuous variables, the Kruskal–Wallis test was applied to compare differences between the groups. Multivariable logistic regression analysis was used to assess the associations between the NLR, PLR, and LMR and delirium. To reduce bias caused by confounders, we adjusted for age, sex, ethnicity, BMI, and alcohol abuse in Model 1. Model 2 was adjusted for age, sex, ethnicity, BMI, alcohol abuse, SOFA score, GCS score, and comorbidities, including hypertension, diabetes mellitus, chronic pulmonary disease, chronic liver disease, chronic renal disease, congestive heart failure, myocardial infarction, peripheral vascular disease, and obstructive sleep apnea. Model 3 was further adjusted for benzodiazepine use and laboratory results, including hemoglobin levels, glucose, albumin, and creatinine, based on Model 2. The results were presented as odds ratios (ORs) with 95% confidence intervals (CIs), and statistical significance was determined at a *p* < 0.05.

In addition, we performed a two-sample MR analysis to re-evaluate the causal relationships between the NLR, PLR, LMR, and delirium. First, we selected single nucleotide polymorphisms (SNPs) that were strongly associated with the NLR, PLR, and LMR, with a *p* < 5 × 10^–8^. In addition, to exclude linkage disequilibrium (LD), we set *R*^2^ to <0.001 (clumping window size = 10,000 kb). Then, the SNPs with a minor allele frequency (MAF) ≤ 0.01 were removed. Finally, we harmonized the alleles of the exposures and outcomes to eliminate ambiguous SNPs with inconsistent alleles and intermediate allele frequencies. For the MR analysis, the inverse-variance weighted (IVW) method was used as the primary analytical approach. The MR-Egger, weighted median, and weighted mode methods were used to validate the IVW results. MR-Pleiotropy RESidual Sum and Outlier (MR-PRESSO) was used to identify potential outliers and horizontal pleiotropy. Cochrane’s Q test and MR-Egger intercept were used to assess heterogeneity and directional pleiotropy, respectively (31, 32). Sensitivity analyses were also performed using leave-one-out analysis. All analyses were two-sided and were performed using the TwoSampleMR and MR-PRESSO packages in R software (version 4.0.2).

## 3 Results

### 3.1 Baseline characteristics

In this study, a total of 1,436 patients with IS were included for analysis, and the selection process is shown in Figure 1. The median age of the included patients was 71 years (IQR: 59–81 years), and 52.4% were male. Among them, 14.9% experienced delirium during hospitalization and 224 patients (15.6%) had fatal events. Table 1 shows the baseline characteristics of the patients, stratified according to the presence or absence of delirium. Compared to the patients without delirium, those with delirium tended to have more comorbidities, higher SOFA scores on admission, a higher proportion of benzodiazepine use, poorer nutritional status, unstable vital signs, and longer hospital stays. The NLR values were significantly higher in the patients with delirium compared to the patients without delirium (7.23 vs. 5.65, *p* = 0.000), while the LMR values were significantly lower in the delirium group (2.22 vs. 2.72, *p* = 0.000). There was no significant difference in the PLR values between the two groups.

**Table 1:** Baseline characteristics (*n* = 1,436).

Variables	Total, *n* = 1,436	No delirium, *n* = 1,222	Delirium, *n* = 214	*p*-value
Age, years, median (IQR)	71 (59, 81)	71 (58, 82)	71 (61, 82)	0.618
Male, *n* (%)	752 (52.4)	629 (51.5)	123 (57.5)	0.105
Ethnicity				0.506
White	933 (65.0)	799 (65.4)	134 (62.6)	
Black	138 (9.6)	115 (9.4)	23 (10.7)	
Asian	31 (2.2)	25 (2.0)	6 (2.8)	
Other	334 (23.3)	283 (23.2)	51 (23.8)	
BMI, kg/m^2^	27.2 (23.6, 31.3)	27.1 (23.5, 31.2)	27.7 (24.2, 32.9)	0.029
Comorbidity, *n* (%)				
Hypertension	1,065 (74.2)	900 (73.6)	165 (77.1)	0.287
Diabetes	459 (32.0)	364 (29.8)	95 (44.4)	0.000
Chronic pulmonary disease	277 (19.3)	234 (19.1)	43 (20.1)	0.747
Chronic liver disease	65 (4.5)	48 (3.9)	17 (7.9)	0.009
Chronic renal disease	304 (21.2)	242 (19.8)	62 (29.0)	0.002
Congestive heart failure	409 (28.5)	323 (26.4)	86 (40.2)	0.000
Myocardial infarction	292 (20.3)	229 (18.7)	63 (29.4)	0.000
Peripheral vascular disease	237 (16.5)	189 (15.5)	48 (22.4)	0.011
Obstructive sleep apnea	110 (7.7)	83 (6.8)	27 (12.6)	0.003
Alcohol abuse	44 (3.1)	37 (3.0)	7 (3.3)	0.849
Use of benzodiazepines	369 (25.7)	301 (24.6)	68 (31.8)	0.027
GCS score	15 (14, 15)	15 (14, 15)	15 (14, 15)	0.129
SOFA score	3 (2, 6)	3 (2, 5)	5 (3, 8)	0.000
Laboratory findings				
Albumin, g/dL	3.7 (3.3, 4.1)	3.8 (3.4, 4.1)	3.6 (3.1, 4.0)	0.001
Creatinine, mg/dL	1.0 (0.8, 1.4)	1.0 (0.8, 1.4)	1.2 (0.9, 1.9)	0.000
Hemoglobin, g/dL	12.5 (10.8, 13.9)	12.6 (10.9, 14.0)	11.8 (10.1, 13.4)	0.000
Glucose, mg/dL	136 (110, 181)	133.0 (109.0, 176.3)	160.5 (127.0, 221.0)	0.000
White blood cell counts, ×10⁹/L	12.1 (9.0, 16.0)	11.8 (8.8, 15.7)	13.1 (9.8, 17.4)	0.000
Platelets, ×10⁹/L	223 (176, 285)	226 (178, 286)	204.5 (159, 277)	0.004
Lymphocytes, ×10⁹/L	1.42 (0.95, 2.01)	1.44 (0.97, 2.03)	1.32 (0.82, 1.87)	0.013
Monocytes, ×10⁹/L	0.55 (0.37, 0.80)	0.54 (0.37, 0.77)	0.62 (0.40, 0.92)	0.002
Neutrophils, ×10⁹/L	8.22 (5.77, 11.76)	8.05 (5.64, 11.61)	9.65 (6.26, 12.60)	0.005
NLR	5.88 (3.40, 9.89)	5.65 (3.25, 9.62)	7.23 (4.18, 11.91)	0.000
PLR	155.24 (103.85, 242.82)	156.12 (103.45, 242.27)	153.46 (107.74, 249.63)	0.810
LMR	2.67 (1.60, 4.12)	2.72 (1.65, 4.28)	2.22 (1.26, 3.40)	0.000
Vital signs on the first day in the ICU				
MAP, mmHg	82 (74, 92)	82 (74, 93)	78 (72, 89)	0.001
Heart rate, beats/min	79 (71, 90)	79 (70, 89)	82 (73, 94)	0.000
Respiration rate, breaths/min	19 (17, 21)	18 (17, 21)	20 (18, 22)	0.000
SpO_2_, %	97 (96, 99)	97 (96, 99)	97 (96, 98)	0.291
Length of ICU stay, days	2.8 (1.5, 6.1)	2.5 (1.4, 5.3)	4.9 (2.2, 8.9)	0.000
Length of hospital stay, days	7.8 (4.2, 14.5)	7.0 (3.9, 11.9)	13.6 (7.8, 23.8)	0.000
Hospital mortality, *n* (%)	224 (15.6)	193 (15.8)	31 (14.5)	0.627

All data were presented as median (interquartile range) or number of patients (%). Abbreviations: BMI, body mass index; SOFA, Sequential Organ Failure Assessment; GCS, Glasgow Coma Scale; NLR, neutrophil-to-lymphocyte ratio; PLR, platelet-to-lymphocyte ratio; LMR, lymphocyte-to-monocyte ratio; MAP, mean arterial blood pressure; SpO_2_, percutaneous oxygen saturation; ICU, Intensive Care Unit.

### 3.2 The association between the NLR, PLR, and LMR, and delirium

**Figure 2:**
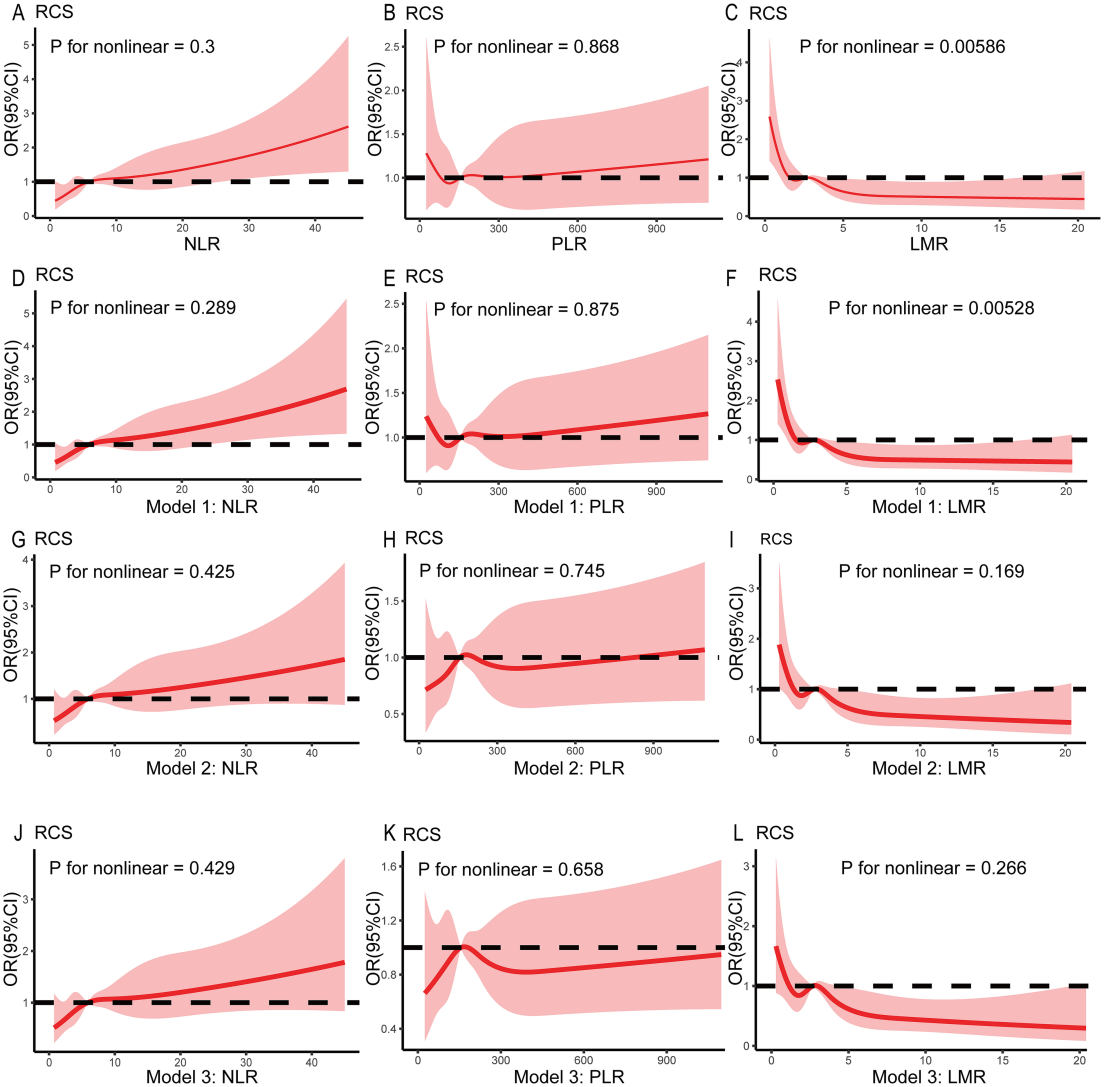
Restricted spline curves showing the relationship between the NLR, PLR, and LMR and delirium in the patients with ischemic stroke. The red bold line denotes the OR, while the shaded area represents the 95% CI. No covariates were adjusted in **(A–C)**. Age, sex, ethnicity, BMI, and lifestyle factors were adjusted in Model 1 **(D–F)**. Model 2 was further adjusted for comorbidities, SOFA score, and GCS score based on Model 1 **(G–I)**. In Model 3, laboratory results and benzodiazepine use were adjusted based on Model 2 **(J–L)**. BMI, body mass index; SOFA, Sequential Organ Failure Assessment; GCS, Glasgow Coma Scale.

Table 2 shows the association of the NLR, PLR, and LMR with the incidence of delirium after IS. In the univariate logistic regression analysis, an increase in the NLR was independently associated with an increased risk of delirium (OR 1.032, 95% CI 1.016–1.049). After adjustment for age, sex, ethnicity, BMI, and lifestyle factors in Model 1, this association remained significant (OR 1.034, 95% CI 1.017–1.050). The relationship between the elevated NLR and delirium risk persisted after adjustment for comorbidities, SOFA score, and GCS score in Model 2 (OR 1.023, 95% CI 1.006–1.041), as well as for laboratory results and benzodiazepine use in Model 3 (OR 1.022, 95% CI 1.004–1.039). However, in the univariate logistic regression analysis, as well as in models 1, 2, and 3, there was no significant association between an increase in the PLR and the risk of delirium (all *p* > 0.05). In the regression analysis between the LMR and delirium, the univariate logistic regression showed that an increase in the LMR was independently associated with a decreased risk of delirium (OR 0.891, 95% CI 0.831–0.956). This significant association remained after adjustment for confounders in Model 1, Model 2, and Model 3 (all *p* < 0.05). In addition, when the NLR was categorized as a nominal variable, the patients in the second, third, and highest quartiles of the NLR were significantly associated with an increased risk of delirium across all three logistic regression models (Model 1, Model 2, and Model 3) compared to those in the lowest quartile (Table 3). Similarly, the patients in the second quartile of the PLR were significantly associated with a higher risk of delirium compared to the patients in the lowest quartile: Model 2 (OR 1.579, 95% CI 1.026–2.429) and Model 3 (OR 1.574, 95% CI 1.019–2.431). Lastly, the patients in the highest quartile of the LMR were negatively associated with an increased risk of delirium compared to the patients in the lowest quartile: Model 1 (OR 0.430, 95% CI 0.276–0.672), Model 2 (OR 0.489, 95% CI 0.310–0.773), and Model 3 (OR 0.503, 95% CI 0.317–0.798). For the NLR and LMR, as well as the PLR in models 2 and 3, the restricted cubic spline (RCS) models showed that they were non-linearly correlated with delirium (all non-linear *p* < 0.05) (Figure 2).

**Table 2:** Multivariable logistic regression analysis of the associations between the NLR, PLR, and LMR and delirium after ischemic stroke.

Variables	OR (95% CI)			
	Crude model	Model 1	Model 2	Model 3
NLR	1.032 (1.016–1.049)*	1.034 (1.017–1.050)*	1.023 (1.006–1.041)*	1.022 (1.004–1.039)*
PLR	1.000 (1.000–1.001)	1.000 (1.000–1.001)	1.000 (1.000–1.001)	1.000(1.000–1.001)
LMR	0.891 (0.831–0.956)*	0.891 (0.830–0.956)*	0.905 (0.847–0.967)*	0.907 (0.850–0.968)*

^*^p < 0.05. Bold values indicate statistically significant results. Crude model: Adjusted for none. Model 1: Adjusted for age, sex, ethnicity, BMI, and alcohol abuse. Model 2: Adjusted for age, sex, ethnicity, BMI, alcohol abuse, SOFA score, GCS score, and comorbidities, including hypertension, diabetes mellitus, chronic pulmonary disease, chronic liver disease, chronic renal disease, congestive heart failure, myocardial infarction, peripheral vascular disease, and obstructive sleep apnea. Model 3: Further adjusted for benzodiazepine use and laboratory results, including hemoglobin levels, glucose, albumin, and creatinine. NLR, neutrophil-to-lymphocyte ratio; PLR, platelet-to-lymphocyte ratio; LMR, lymphocyte-to-monocyte ratio; CI, confidential interval; OR, odds ratio.

**Table 3:** Odds ratio (95% CI) for delirium across the quartiles of the NLR, PLR, and LMR.

Variables	OR (95% CI)				P for trend
	Quartile 1	Quartile 2	Quartile 3	Quartile 4	
NLR					
Model 1	Reference	1.933 (1.210–3.085)*	1.982 (1.239–3.171)*	2.650 (1.679–4.182)*	**0.001**
Model 2	Reference	1.715 (1.062–2.769)*	1.748 (1.077–2.837)*	2.135 (1.325–3.440)*	**0.02**
Model 3	Reference	1.733 (1.072–2.804)*	1.745(1.069-2.847)*	2.080 (1.282–3.375)*	**0.028**
PLR					
Model 1	Reference	1.305 (0.865–1.969)	1.054 (0.688–1.615)	1.197 (0.787–1.821)	0.574
Model 2	Reference	1.579 (1.026–2.429)*	1.274 (0.814–1.992)	1.378(0.890–2.134)	0.213
Model 3	Reference	1.574 (1.019–2.431)*	1.245 (0.793–1.955)	1.252 (0.803–1.952)	0.239
LMR					
Model 1	Reference	0.814 (0.553–1.197)	0.701 (0.471–1.042)	0.430(0.276–0.672)*	**0.003**
Model 2	Reference	0.911 (0.609–1.362)	0.871(0.573–1.324)	0.489(0.310–0.773)*	**0.016**
Model 3	Reference	0.957 (0.637–1.438)	0.954 (0.623–1.460)	0.503(0.317–0.798)*	**0.017**

^*^p < 0.05. Bold values indicate statistically significant results. Model 1: Adjusted for age, sex, ethnicity, BMI, and alcohol abuse. Model 2: Adjusted for age, sex, ethnicity, BMI, alcohol abuse, SOFA score, GCS score, and comorbidities, including hypertension, diabetes mellitus, chronic pulmonary disease, chronic liver disease, chronic renal disease, congestive heart failure, myocardial infarction, peripheral vascular disease, and obstructive sleep apnea. Model 3: Further adjusted for benzodiazepine use and laboratory results, including hemoglobin levels, glucose, albumin, and creatinine. NLR, neutrophil-to-lymphocyte ratio; PLR, platelet-to-lymphocyte ratio; LMR, lymphocyte-to-monocyte ratio; CI, confidential interval; OR, odds ratio.

### 3.3 Subgroup analysis

**Figure 3:**
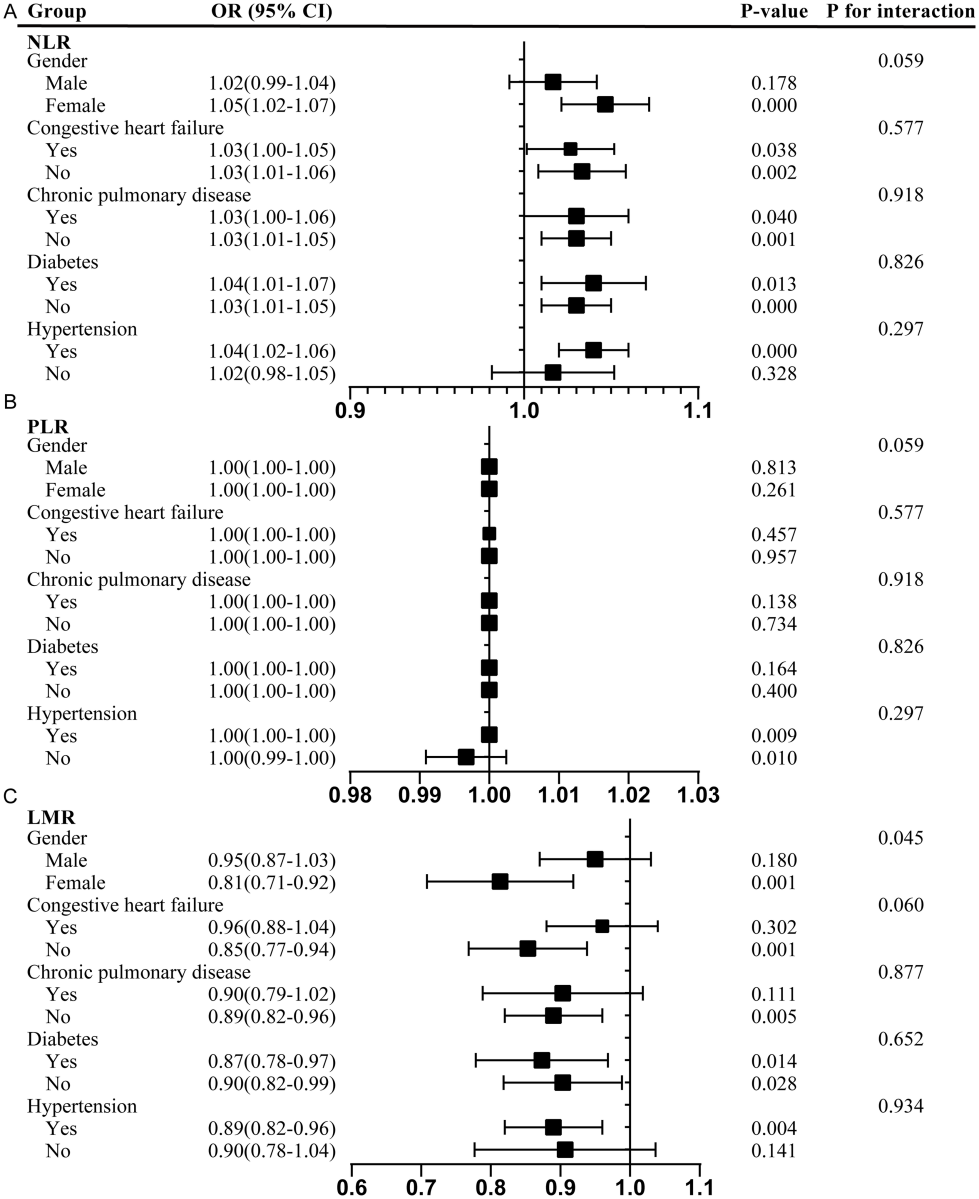
Subgroup analyses of the association between the NLR, PLR, and LMR and delirium. **(A)** NLR; **(B)** PLR; **(C)** LMR. NLR, neutrophil-to-lymphocyte ratio; PLR, platelet-to-lymphocyte ratio; LMR, lymphocyte-to-monocyte ratio; OR, odds ratios; CI, confidence interval.

In addition, subgroup analyses were conducted to assess the associations between the NLR, PLR, and LMR and delirium across different populations. The participants were stratified based on sex, congestive heart failure, chronic pulmonary disease, hypertension, and diabetes. The results are presented in Figure 3.

The positive correlation between the NLR and delirium was particularly pronounced in the female patients (OR 1.05, 95% CI 1.02–1.07) and the participants with hypertension (OR 1.04, 95% CI 1.02–1.06). This association remained consistent across the subgroups with and without congestive heart failure, chronic pulmonary disease, and diabetes, highlighting potential sex-specific and comorbidity-dependent influences. However, there was a positive association between the PLR and delirium in those with hypertension (OR 1.00, 95% CI 1.00–1.00) and a negative association in those without hypertension (OR 1.00, 95% CI 0.99–1.00). In contrast, the LMR showed a significant negative association with delirium, particularly in the female participants (OR 0.81, 95% CI 0.71–0.92), participants without congestive heart failure (OR 0.85, 95% CI 0.77–0.94), participants without chronic pulmonary disease (OR 0.89, 95% CI 0.82–0.96), participants with hypertension (OR 0.89, 95% CI 0.82–0.96), and those with and without diabetes. The interaction test suggested that the relationship between the LMR and delirium was affected by sex (male/female) (*Pp* < 0.05). Female individuals may serve as effect modifiers (Figure 3).

**Figure 4:**
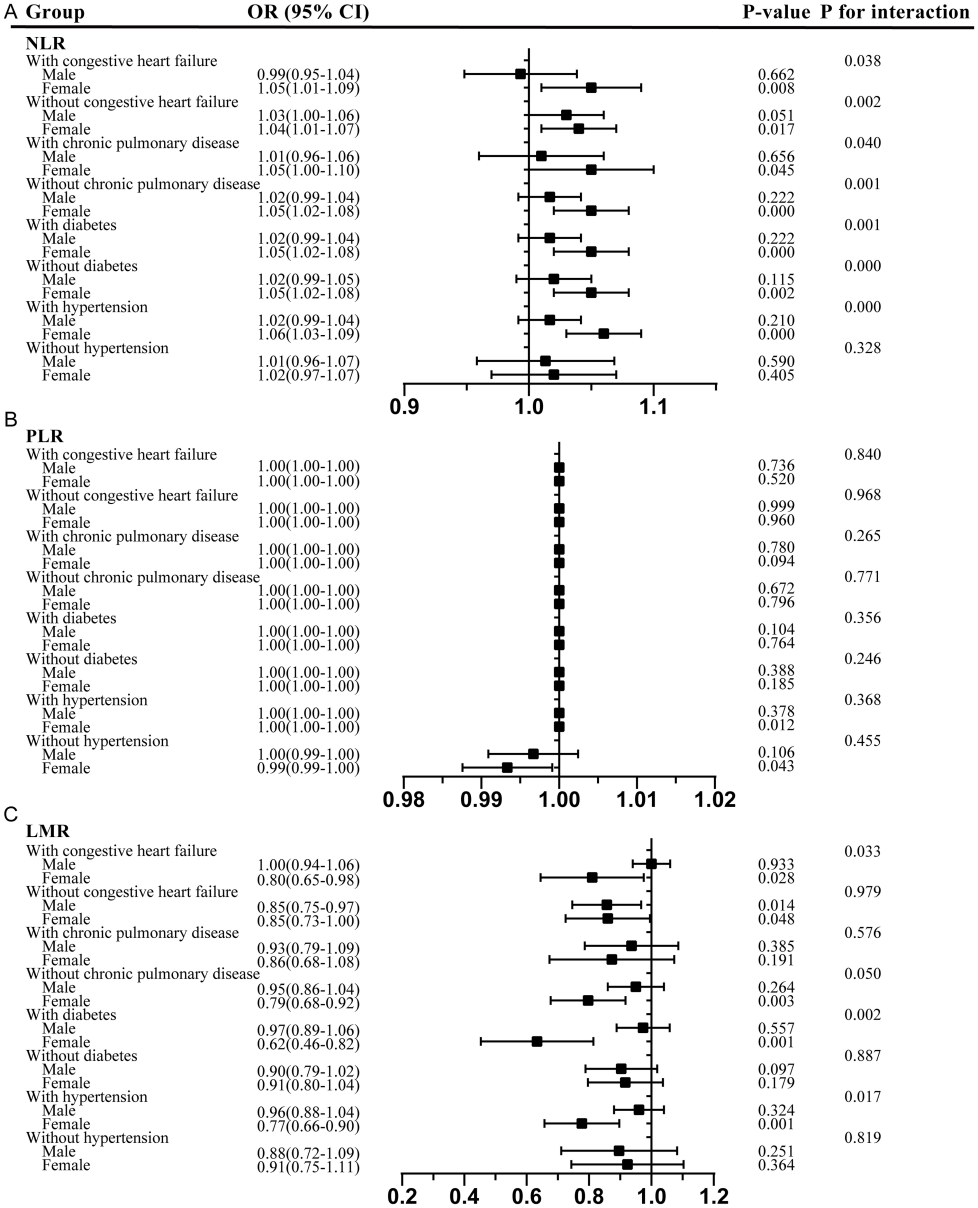
Sex-stratified analysis of comorbidities in the association between the NLR, PLR, and LMR and delirium. **(A)** NLR; **(B)** PLR; **(C)** LMR. NLR, neutrophil-to-lymphocyte ratio; PLR, platelet-to-lymphocyte ratio; LMR, lymphocyte-to-monocyte ratio; OR, odds ratios; CI, confidence interval.

Moreover, in the sex-stratified analysis of comorbidities, the positive correlation between the NLR and delirium was more pronounced in the female participants across all comorbidity subgroups, including those with and without congestive heart failure, chronic pulmonary disease, and diabetes, as well as those with hypertension (Figure 4). The interaction test suggested that sex played a pivotal role in modulating these relationships, with female individuals acting as potential effect modifiers (*p* < 0.05). Similarly, the negative association between the PLR and delirium was more evident in the female participants, with and without hypertension. Furthermore, the LMR showed a significant protective effect against delirium in the female participants across all comorbidity subgroups, including those with congestive heart failure, chronic pulmonary disease, diabetes, and hypertension.

### 3.4 Causal association between the NLR, PLR, and LMR and delirium in MR

In the MR study, we screened a total of 59, 85 and 44 SNPs, to investigate the causal relationship between the NLR, PLR, and LMR and delirium. Detailed information about these SNPs can be found in Supplementary Table S2. The results showed that an increase in the genetically predicted PLR was causally associated with a decreased risk of delirium (IVW: OR 0.996, 95% CI 0.994–0.999, *p* = 0.040). This finding was further validated by MR-PRESSO (OR 0.997, 95% CI 0.994–0.999, *p* = 0.036) (Table 4). On the other hand, no significant association was observed between an increase in the genetically predicted NLR (IVW: OR 0.799, 95% CI 0.628–1.017, *p* = 0.068) and LMR (IVW: OR 1.033, 95% CI 0.942–1.133, *p* = 0.489) and the risk of delirium. These results indicated a lack of evidence for a causal relationship between the NLR and LMR levels and the risk of delirium. In addition, the intercept obtained from the MR-Egger regression was not significant (Supplementary Table S3), suggesting that there was no significant horizontal pleiotropy in the MR analysis. The results of the MR-PRESSO heterogeneity test further confirmed the accuracy of the MR-Egger regression. In the absence of heterogeneity and pleiotropy, the abovementioned MR results were considered reliable.

**Table 4:** The results of the MR study investigating the effects of the NLR, PLR, and LMR on delirium.

Exposure	Methods	nSNP	OR	95% CI	*p*-value
NLR	IVW	59	0.799	0.628–1.017	0.068
	Weighted median	59	0.991	0.699–1.404	0.958
	MR-Egger	59	1.406	0.704–2.810	0.339
	MR-PRESSO	59	0.799	0.628–1.017	0.074
PLR	IVW	85	0.996	0.994–0.999	0.04*
	Weighted median	85	0.998	0.993–1.002	0.314
	MR-Egger	85	1.005	0.996–1.013	0.276
	MR-PRESSO	85	0.997	0.994–0.999	0.036*
LMR	IVW	44	1.033	0.942–1.133	0.489
	Weighted median	44	1.031	0.898–1.184	0.661
	MR-Egger	44	1.011	0.815–1.254	0.922
	MR-PRESSO	44	1.033	0.945–1.130	0.478

^*^Represents that the *p*-value was significant. NLR, neutrophil-to-lymphocyte ratio; PLR, platelet-to-lymphocyte ratio; LMR, lymphocyte-to-monocyte ratio; CI, confidential interval; IVW, inverse-variance weighted; MR-PRESSO, Mendelian Randomization Pleiotropy RESidual Sum and Outlier; OR, odds ratio; SNP, single nucleotide polymorphism.

In the sensitivity analysis, the funnel plots (Supplementary Figure S1) showing the results of the MR analysis displayed a symmetrical shape, indicating no heterogeneity in the results. The leave-one-out analysis revealed a significant association between the NLR and delirium when the SNPs “rs61839660,” “rs77919370,” “rs4760,” “rs11975539,” “rs6981399,” and “rs4792711” were excluded from the model (Supplementary Figure S2). However, this association was no longer significant when these SNPs were included. In the analysis of the association between the PLR and delirium, excluding the SNPs “rs6713632,” “rs4907923,” “rs1982151,” “rs7003580,” “rs1076415,” “rs10794175,” “rs11953668,” “rs10843375,” “rs80230005,” “rs536327,” and “rs6679677” resulted in no significant association between the PLR and delirium, whereas the association became significant when these SNPs were included (Supplementary Figure S3). The association between the LMR and delirium was not influenced by any single SNP (Supplementary Figure S4).

## 4 Discussion

In this study, we investigated the association between the NLR, PLR, and LMR and the occurrence of delirium in IS patients admitted to the ICU. We found that 214 (14.9%) of the 1,436 IS patients developed delirium. High levels of the NLR and PLR were associated with an increased risk of delirium during hospitalization, and low levels of the LMR were associated with an increased risk of delirium during hospitalization. This association persisted after adjusting for age, sex, ethnicity, comorbidities, and poor lifestyle factors. However, after further adjustment for other study outcomes, vital signs, and disease severity scores, the association between the high NLR and PLR values and the risk of delirium disappeared. In contrast, the increased risk of delirium associated with the low LMR values remained. Furthermore, in the MR analysis, we found no causal relationship between either the NLR or LMR and the occurrence of delirium, while a significant relationship between the PLR and delirium persisted.

Delirium is an acute state of cerebral dysfunction characterized by sudden, fluctuating disturbances in consciousness and impaired attention (33–35). Increasing evidence suggests a strong association between delirium and adverse patient outcomes. Patients with delirium face a 10-fold increased risk of death, a 3–5-fold increased risk of complications, longer hospital stays, and a greater need for post-discharge care and support measures (33). In addition, patients with delirium are more likely to experience poor functional and cognitive recovery. The pathophysiological mechanisms underlying delirium are not fully understood (36). Currently, there are no effective medications or interventions for the treatment of delirium. Therefore, early identification of patients at risk of delirium is important (36).

Numerous studies have examined the association between inflammatory cytokines and delirium, with some showing that levels of inflammatory mediators such as plasma interleukin (IL)-6, C-reactive protein (CRP), IL-8, IL-10, and tumor necrosis factor (TNF)-*α* are significantly elevated in patients with delirium (14). However, the relationship between the NLR, PLR, and LMR and delirium in IS patients remains underexplored.

Compared to cytokines and acute-phase reactants, neutrophils, lymphocytes, and platelet counts are routinely measured as part of the hospital admission laboratory test for the majority of patients, with low acquisition costs and minimal requirements (37). In addition, these blood cell ratios reflect the dynamic balance between pro-inflammatory and anti-inflammatory immune responses, which are believed to contribute directly to the pathophysiology of delirium via neuroinflammatory mechanisms. As a result, they may offer greater clinical utility in predicting delirium after IS, facilitating early intervention to reduce its incidence.

In various inflammatory conditions, the NLR has been positively correlated with IL-6 levels, which supports its potential as a reliable marker of systemic inflammation (38–40). Neutrophils, as key pro-inflammatory immune cells, are elevated in response to acute inflammatory processes, indicating an intense inflammatory response. Conversely, lymphocytes typically play a suppressive role in inflammation, and their reduced numbers may indicate immune suppression. Thus, an elevated NLR value is generally associated with increased inflammation or immune system dysregulation. Similarly, the PLR has been associated with elevated levels of TNF-*α* in patients experiencing acute inflammation, underscoring its role as an inflammatory marker (41, 42). In addition to their traditional role in coagulation, platelets also contribute to immune responses by promoting pro-inflammatory pathways. The elevation of platelets and the corresponding decrease in lymphocytes observed in an increased PLR may signal a systemic inflammatory response, further associating this ratio with acute or chronic inflammatory conditions. Monocytes, which play a critical role in immune modulation, particularly in chronic inflammation, contribute to the regulation of immune responses. A low LMR value generally reflects a state of immune suppression, while a higher LMR value suggests a more balanced immune environment or a dominant anti-inflammatory response. This suggests that the LMR could offer insights into immune balance in IS patients, potentially providing prognostic value in predicting delirium risk.

These findings indicate that the NLR, PLR, and LMR may reflect similar inflammatory processes as traditional markers, reinforcing their clinical utility in predicting delirium after IS. Although IL-6 and TNF-*α* are well-established markers of inflammation, the NLR, PLR, and LMR offer a more accessible and cost-effective alternative for the assessment of inflammatory status in acute conditions such as IS (43). Moreover, the NLR, PLR, and LMR have demonstrated strong prognostic value in a variety of diseases, including IS (44), cardiovascular events (45), and cancer (46).

The pathophysiological mechanisms underlying delirium remain unclear. One hypothesis is that neuroinflammation-induced metabolic dysregulation contributes to the occurrence of delirium. Infection, surgery, or trauma can trigger acute peripheral inflammation in the body, leading to increased adherence and activation of leukocytes on the endothelial cells of cerebral blood vessels. This results in the significant release of free radicals and enzymes, which, in turn, disrupt the integrity of the endothelial cell membrane and intercellular adhesion, increase the permeability of the blood–brain barrier, cause perivascular edema, reduce perfusion, and increase the oxygen diffusion distance, ultimately leading to neuronal hypoxia and the manifestation of delirium (47–49). In addition, the elderly or those with impaired cognitive function are more susceptible to delirium (50). This increased susceptibility may be due to age-related changes in neurotransmitters, decreased cerebral blood flow, neuronal loss, and reduced intracellular signaling capacity. When stimulated by factors such as peripheral inflammation, cognitive decline is further exacerbated, leading to the manifestation of delirium.

Our findings highlight the relevance of systemic inflammatory biomarkers—NLR, PLR, and LMR—in the context of delirium. In the occurrence and prognosis of delirium, variations in these indicators reflect the body’s inflammatory response, immune dysfunction, cytokine release, and other related processes.

In addition to the overall analysis, we performed subgroup analyses based on sex and comorbidities, which provided further insights into the relationship between the inflammatory markers and delirium. Our subgroup analysis suggested that sex may influence the association between the inflammatory markers and delirium. Specifically, the positive correlation between the NLR and delirium was more pronounced in the female patients, while the LMR appeared to offer a protective effect in this group. These findings imply that sex may modulate the inflammatory response and its impact on delirium. One possible explanation for these sex-related differences is the role of estrogen in modulating immune responses, including leukocyte proliferation and antibody production, which may result in a stronger immune response in female individuals, thereby influencing the relationship between inflammation and delirium (51).

Moreover, our subgroup analysis highlighted the role of comorbidities (such as hypertension, congestive heart failure, and chronic pulmonary disease) in modulating the relationship between the inflammatory markers and delirium. In the hypertensive patients, we observed a significant positive correlation between the NLR and delirium, along with a negative correlation between the LMR and delirium. Hypertension is a well-established risk factor for IS, exerting chronic effects on vascular health and the blood–brain barrier integrity through increased shear stress, endothelial dysfunction, and large artery stiffness (52, 53). In hypertensive individuals, the increase in systemic inflammation may further exacerbate endothelial dysfunction, leading to an elevated risk of delirium. In this context, the hypertensive state may amplify the inflammatory response, thereby strengthening the association between the inflammatory markers and delirium in this subgroup.

The present study has several strengths. First, the cohort size was larger than in previous studies. Second, we evaluated the overall systemic inflammation of the patients using the NLR, PLR, and LMR as inflammatory markers, which are simple and easily obtainable methods. Third, we adjusted for more confounders than previous studies, including laboratory indicators and vital signs, to determine the association between the NLR, PLR, and LMR and delirium. Finally, we performed an MR analysis to investigate the causal relationship between these markers and delirium at the genetic level.

However, there are also some limitations to this study. First, the definition of delirium in the FinnGen database (ID: F5_DELIRIUM) encompasses various etiologies and is not limited to IS-related delirium. This phenotypic heterogeneity could have potentially diluted the specificity of our findings in the MR analysis. Second, IS patients often present with acute and severe neurological impairments, which may hinder the recognition of delirium, potentially leading to an underestimation of its true prevalence. Third, the NLR, PLR, and LMR data used in the MR analysis were derived from secondary GWAS analyses, which might have introduced errors or biases. The inconsistency between our observational study and the MR study suggests that the NLR, PLR, and LMR remain valuable predictors of post-IS delirium and require further rigorous exploration, such as prospective studies.

## 5 Conclusion

In conclusion, our observational study showed that the NLR, PLR, and LMR were significantly associated with the occurrence of delirium and that the inclusion of the NLR, PLR, and LMR in the delirium prediction model may improve its predictive accuracy. However, further MR analysis revealed a causal relationship only between the PLR and the risk of delirium. Therefore, well-designed prospective cohort studies are warranted to validate the associations between the NLR, PLR, and LMR and delirium, as well as to explore the underlying mechanisms.

## Data Availability

The original contributions presented in this study are included in the article and its Supplementary Material. Further inquiries regarding the data can be directed to the corresponding authors.

## Supplementary material

Supplementary Figure S1 Funnel plots of the MR analyses for the association of NLR, PLR, and LMR and delirium. **(A)** Causal effect of NLR on delirium; **(B)** Causal effect of PLR on delirium; **(C)** Causal effect of LMR on delirium. SNP, single nucleotide polymorphisms; MR, Mendelian randomization.

Supplementary Figure S2 Leave-one-out plots of the MR analyses (IVW model) for the association of NLR and delirium.

Supplementary Figure S3 Leave-one-out plots of the MR analyses (IVW model) for the association of PLR and delirium.

Supplementary Figure S4 Leave-one-out plots of the MR analyses (IVW model) for the association of LMR and delirium.
