# Supplementary figures and images for: The association between the neutrophil-to-lymphocyte ratio, platelet-to-lymphocyte ratio, and lymphocyte-to-monocyte ratio and delirium in ischemic stroke patients
Source: Front Med (Lausanne). 2025 Jan 6;11:1456742. doi: 10.3389/fmed.2024.1456742 (PMC11743177; doi:10.3389/fmed.2024.1456742)

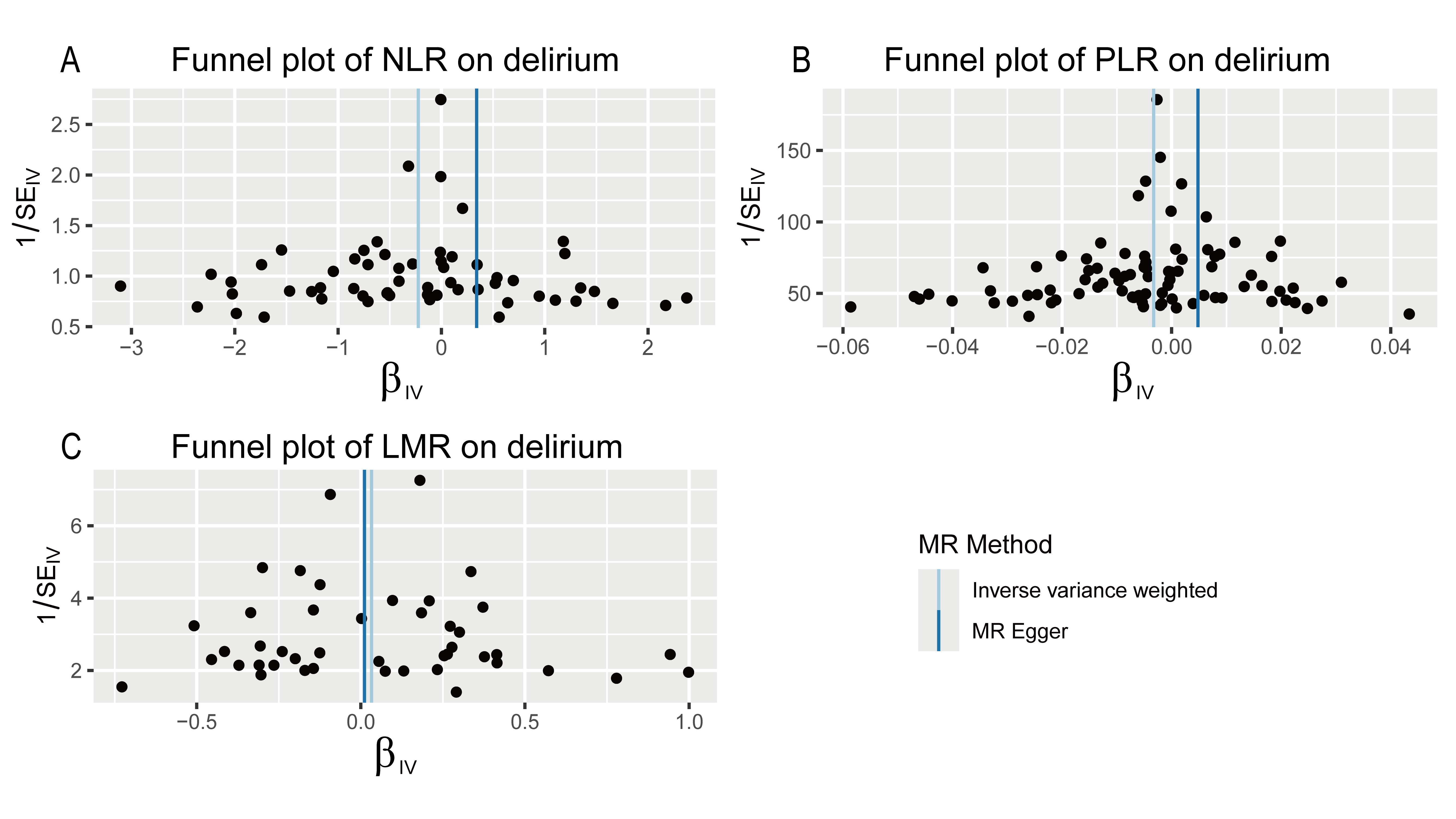

Supplement: Supplementary Figure S1 — Funnel plots of the MR analyses for the association of NLR, PLR, and LMR and delirium. (A) Causal effect of NLR on delirium; (B) Causal effect of PLR on delirium; (C) Causal effect of LMR on delirium. SNP, single nucleotide polymorphisms; MR, Mendelian randomization. [file Image_1.TIF]

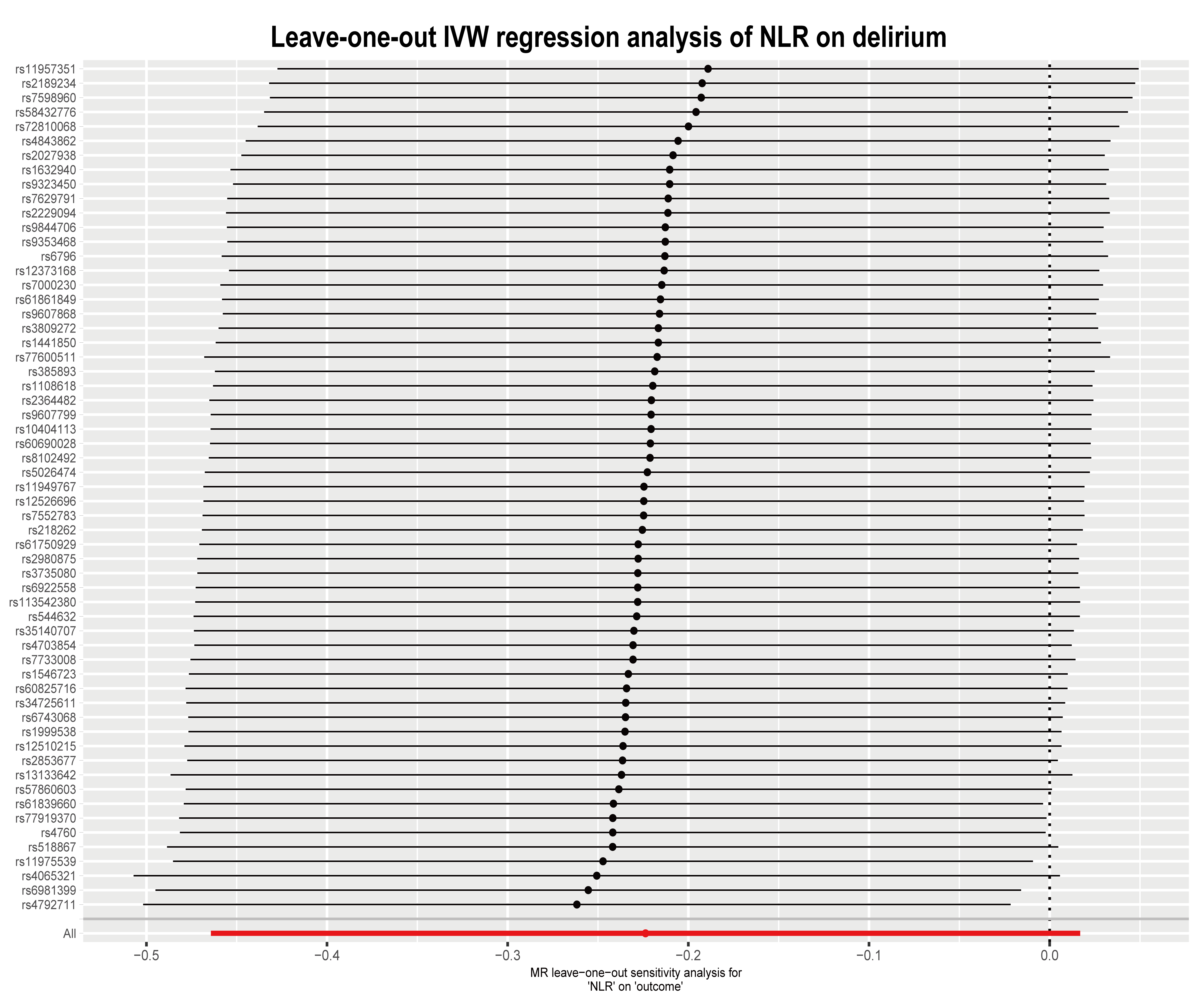

Supplement: Supplementary Figure S2 — Leave-one-out plots of the MR analyses (IVW model) for the association of NLR and delirium. [file Image_2.TIF]

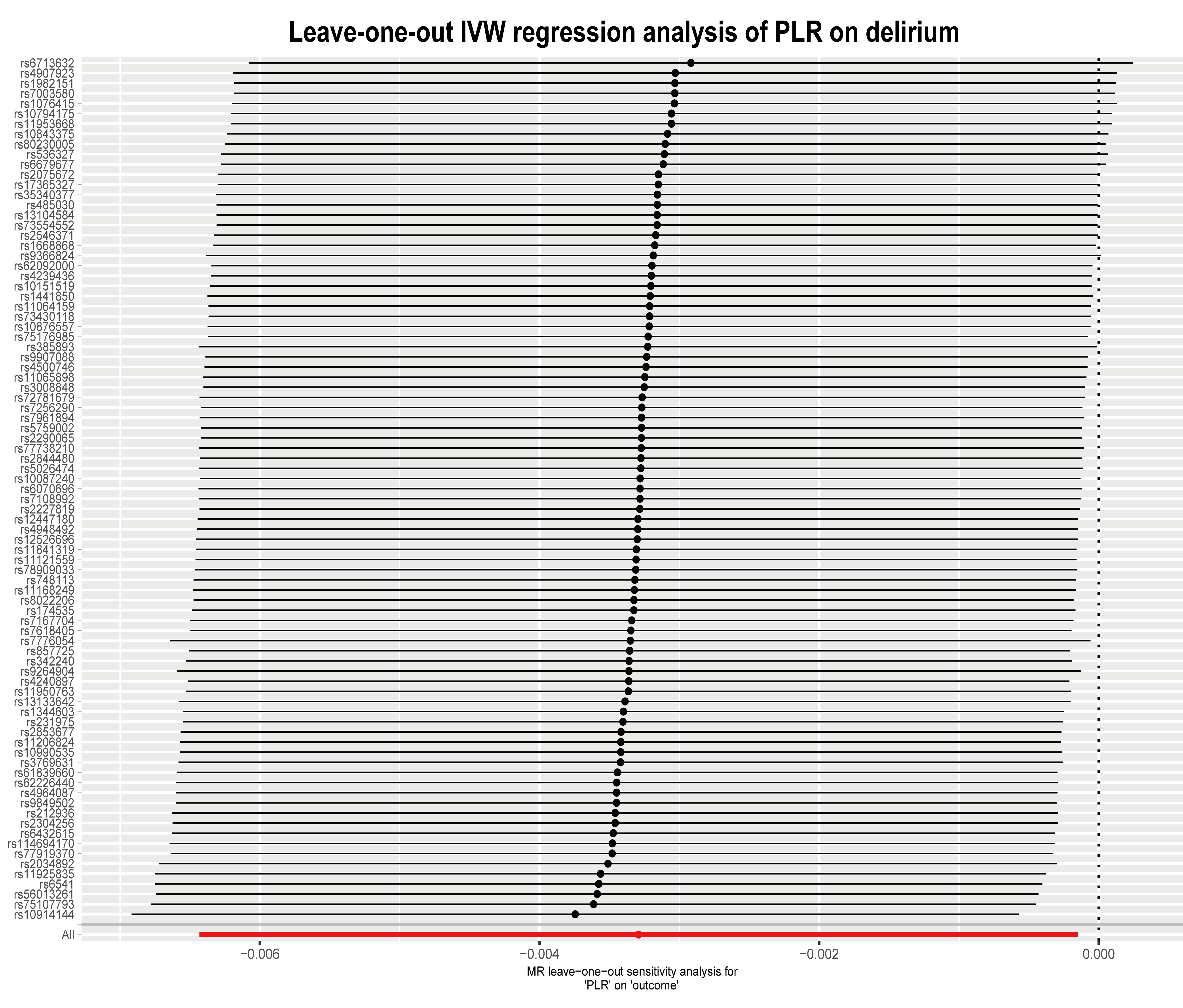

Supplement: Supplementary Figure S3 — Leave-one-out plots of the MR analyses (IVW model) for the association of PLR and delirium. [file Image_3.TIF]

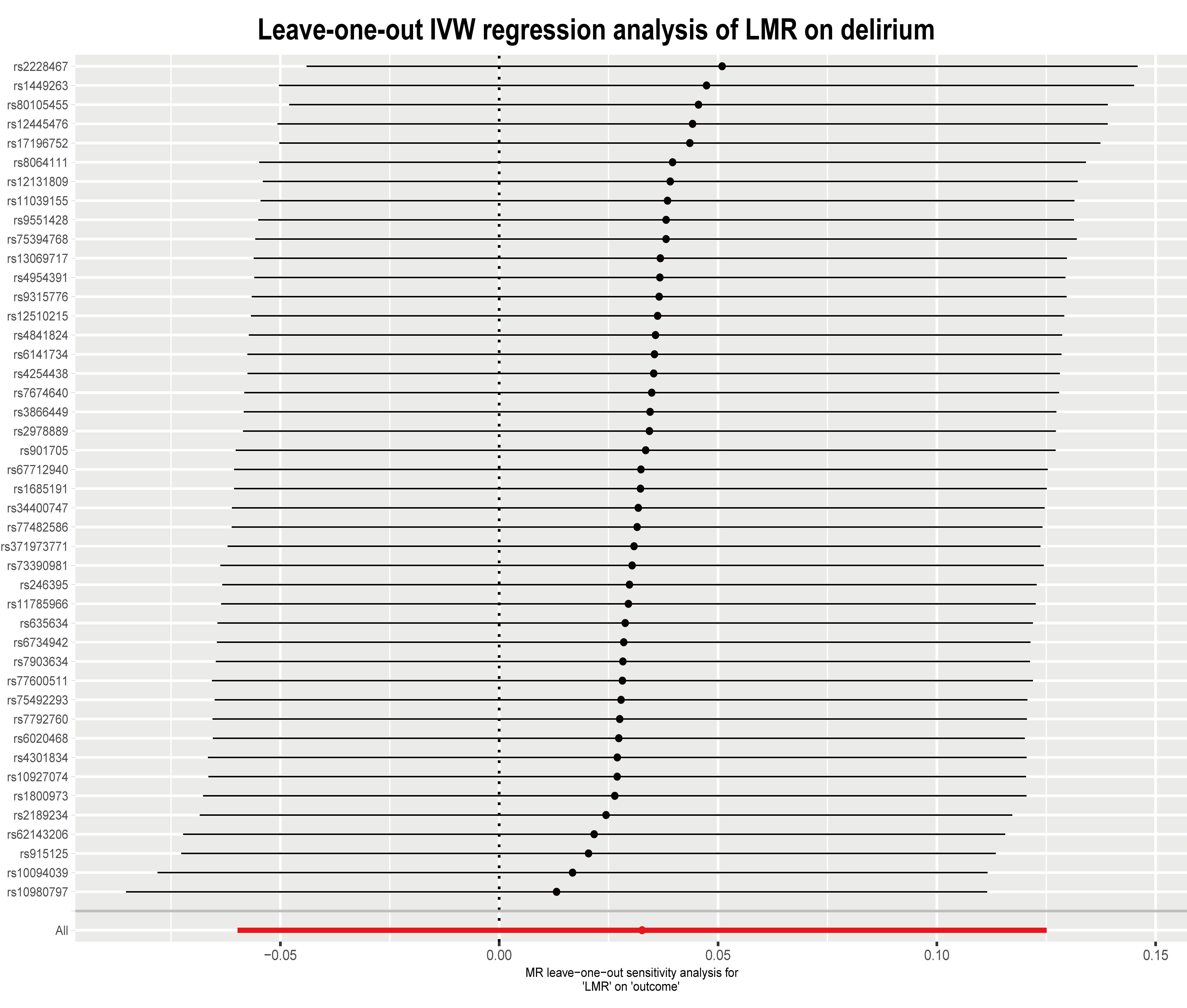

Supplement: Supplementary Figure S4 — Leave-one-out plots of the MR analyses (IVW model) for the association of LMR and delirium. [file Image_4.TIF]
